# QUALITY OF REHABILITATION CARE IN PORTUGUESE STROKE UNITS: FINDINGS FROM 2017–2018 AND 2023 NATIONAL CROSS‑SECTIONAL SURVEYS

**DOI:** 10.2340/jrm.v58.44855

**Published:** 2026-03-29

**Authors:** Joana T. SARMENTO, Ana ALVES, Paulo CASTRO-CHAVES, Bárbara M. CRUZ, Cristina JÁCOME

**Affiliations:** 1 Department of Medicine, Faculty of Medicine, University of Porto, Porto, Portugal; 2 Department of Physical and Rehabilitation Medicine, Unidade Local de Saúde do Médio Ave, Vila Nova de Famalicão; 3 Physical and Rehabilitation Clinic of S. Nicolau, Porto, Portugal; 4 RISE-Health, Faculty of Medicine, University of Porto, Porto, Portugal; 5 Department of Internal Medicine, Unidade Local de Saúde de São João, Porto, Portugal; 6 Department of Physical and Rehabilitation Medicine, Centro Hospitalar Universitário Santo António, Porto, Portugal

**Keywords:** guideline adherence, health services research, quality indicators, rehabilitation, stroke, stroke units

## Abstract

**Objective:**

To evaluate adherence to stroke rehabilitation guidelines in Portuguese stroke units from the physicians’ perspective and examine changes over 6 years.

**Design:**

A national cross-sectional survey across 2 time periods (2017–2018 and 2023).

**Methods:**

Stroke units recognized by the Portuguese Stroke Society were invited to participate: 27 in 2017–2018 and 35 in 2023. A structured questionnaire, aligned with national and international guidelines, assessed 5 domains: team composition, care coordination, early assessment and planning, dysfunction assessments, and post-discharge planning.

**Results:**

Response rates were 93% (*n* = 25) and 80% (*n* = 28). Most units (> 75%) had a physiatrist, physiotherapist, speech therapist, rehabilitation nurse, and social worker; only 20% had the full recommended team. Weekly stroke unit meetings were stable (88–89%), while rehabilitation team meetings increased markedly between the 2 periods (20% vs 72%). Rehabilitation started earlier on weekdays (89% vs 79%) than at weekends (68% vs 57%). Dysphagia screening was common (96% vs 89%), but neurogenic bladder assessment was rare (< 8%). The Modified Rankin Scale and Barthel Index were consistently used. Post-discharge planning remained high (92% vs 89%), with improved coordination between teams (48% vs 89%).

**Conclusion:**

Portuguese Stroke Units demonstrated moderate-to-high adherence to rehabilitation guidelines, with progress in teamwork communication and care coordination, although important gaps remain.

## LAY ABSTRACT

Stroke is one of the leading causes of disability and death in Portugal. Rehabilitation is essential to help people recover after a stroke and to improve their quality of life. To understand how stroke rehabilitation is organized in Portugal, we carried out 2 national surveys, in 2017–2018 and in 2023, covering all official stroke units. We asked about rehabilitation teams, care coordination, early assessments, and discharge planning. Most units had key professionals, such as physiatrists, physiotherapists, nurses, speech therapists and social workers, but only a few had the full recommended team, especially lacking occupational therapists and psychologists. Rehabilitation usually started quickly, although less often at weekends. Screening for swallowing problems was common, but bladder problems were rarely assessed. Planning for rehabilitation after hospital discharge was frequent and has improved. These results show progress but also highlight gaps that need attention to ensure better recovery for patients with stroke.

Stroke is the second leading cause of disability and death worldwide, and the first cause of both in Portugal (1). Forecasts for Europe from 2017 to 2047 predict a 17% decrease in stroke mortality, but a 27% increase in its prevalence (2). Estimates for Portugal suggest a slight downward trend in prevalence, declining from 192,300 cases (95% CI: 188,700–196,000) in 2017 to a projected 183,100 (95% CI: 177,300–189,100) by 2027 (2). Improvements in acute-phase treatments have reduced mortality and the severity of disability (3), yet stroke remains in the top 10 causes of disability-adjusted life years (DALYs) worldwide (4). Rehabilitation therefore plays a critical role in stroke patients’ recovery, significantly contributing to improved health outcomes and quality of life (5–7). As stroke prevalence increases, the demand for high-quality rehabilitation services is expected to rise (7).

Interest in the quality of stroke healthcare has increased rapidly around the world (8). European and North America healthcare organizations produced guidelines to measure the quality of care and certify stroke units (9–14). Building on this progress, international guidelines for stroke rehabilitation have been developed to reduce variability in rehabilitation care delivery (5–7, 15–17). Despite organized services in most European countries, large variability persists in the practical application of guidelines and adherence to quality indicators (18). Adherence to these guidelines has consistently been correlated with significant improvements in functional outcomes (19, 20) and lowering of 7-day in-hospital mortality (21).

In Portugal, an evidence-based Standard Clinical Guideline for Stroke Rehabilitation for the Portuguese National Health Service has been in place since 2011 (17, 22) and aligns with major international recommendations ensuring consistency with global standards of care. This guideline outlines quality indicators and timings for stroke rehabilitation, with particular emphasis on the acute phase. It defines standards for early clinical and functional assessment, individualized treatment planning and continuity of care after discharge (17). Additionally, it specifies the appropriate rehabilitation unit following hospital discharge, and the use of standardized functional assessment scales (17).

Although both international and national guidelines have been in place for at least 14 years, and it is acknowledged that adherence to stroke rehabilitation guidelines is crucial for improving patient outcomes, no formal evaluation of adherence to these guidelines or its evolution over time has been conducted in Portugal.

Therefore, this study aimed to evaluate adherence to stroke rehabilitation guideline in Portuguese stroke units from the physicians’ perspective and examine changes over a 6-year period.

## METHODS

### Study design

This was a 2-period cross-sectional study using a structured survey to evaluate structural and process quality indicators of rehabilitation care in Portuguese stroke units. The survey was applied in 2 periods 6 years apart: the first from September 2017 to January 2018, the second in May 2023. The longer duration of the first data collection was due to the face-to-face application of the survey in each unit, while the second round was conducted through an online questionnaire, allowing for a more concentrated collection period (see below). Henceforth, these 2 periods will be referred to as Assessment 1 (A1) and Assessment 2 (A2). The study protocol was reviewed by the Health Ethics Committee of Unidade Local de Saúde do Médio Ave, which raised no ethical concerns and granted a waiver. Study reporting followed the Strengthening the Reporting of Observational Studies in Epidemiology (STROBE) Statement (23).

### Participating centres

All officially recognized Portuguese stroke units were invited: 27 in 2017–2018 (A1) and 35 in 2023 (A2), as listed in the Portuguese Stroke Society’s 2017 and 2021 Stroke Unit Guides (24, 25). Between 2019 and 2021, the 35 stroke units recorded approximately 374 admissions per year, compared with a total institutional average of ~595 stroke admissions (24). This indicates that only ~60% of all stroke patients were treated within a specialized stroke unit, with the remainder managed in other hospital wards (24). The Portuguese Stroke Society periodically assesses overall performance of stroke units in Portuguese hospitals in line with European Stroke Organization guidelines and publishes a guide listing those that meet the essential inclusion criteria. Those evaluations focus on general inclusion criteria rather than on rehabilitation practices. Therefore, we conducted an independent survey focused specifically on rehabilitation guideline adherence. No exclusion criteria were applied.

### Surveys

A structured survey was developed to collect data on characteristics of stroke unit rehabilitation care, based on literature review of national and international guidelines (5, 6, 15–17) (Table SI). Three physiatrists with over 7 years of experience in stroke rehabilitation developed the survey, which was reviewed by 3 additional physiatrists with more than 6 years of stroke rehabilitation experience to test its feasibility.

The questionnaire covered 5 domains: (*i*) multidisciplinary rehabilitation team composition, (*ii*) coordination of care, (*iii*) early assessment and individualized rehabilitation planning, (*iv*) specific dysfunctions assessments (dysphagia and neurogenic bladder), (*v*) post-discharge rehabilitation planning.

Team composition was evaluated by the presence of the diverse health professionals in the rehabilitation team according to the definition of the European Stroke Organization (ESO), the international standards from North America, including both the American Heart Association/American Stroke Association (AHA/ASA), and the Canadian Stroke Best Practice Recommendations, comprising physiatrist, rehabilitation nurse, physiotherapist, occupational therapist, speech and language therapist, nutritionist, social worker, and (neuro)psychologist (9).

Care coordination was assessed through the occurrence of 2 different weekly meetings: (*i*) stroke unit meeting, primarily focused on acute medical management and patient stabilization, involving mainly physicians and the nursing and therapists staff who manage the ward’s daily clinical operations; and (*ii*) the rehabilitation team meeting, involving all members of the multidisciplinary rehabilitation team (physiatrist, rehabilitation nurses, physiotherapists, occupational therapists, speech and language therapists, nutritionists, social workers, and neuropsychologists). Its primary objective is the functional assessment of the patient, the establishment of individualized rehabilitation goals, the coordination of the interprofessional treatment plan, and the definition of a post-discharge rehabilitation plan with guidance for the patient and their family.

Early assessment referred to the initial evaluation by the physiatrist, who coordinated care with the team. Dysphagia and neurogenic bladder assessment referred to timely screening of these conditions. Post-discharge planning was evaluated by the physiatrist’s early definition of rehabilitation needs and referral destination.

Two additional questions on post-discharge planning, adapted to the Portuguese context, were included. After hospital discharge, patients are referred to inpatient or outpatient units according to disability severity, as outlined in the Standard Clinical Guideline for Stroke Rehabilitation (17). Inpatient care may involve Continued Care Units or Rehabilitation Centres, the latter being the most specialized, with the physiatrist responsible for referral decisions. All hospitals also have Discharge Management Teams (DMTs), which coordinate discharges for all conditions, not specifically stroke, to Continued Care Units. In practice, our survey sought to identify whether discrepancies existed between the 2 referral sources.

The survey was used in both assessment periods (A1 and A2). An additional open-ended question was included in the A1 to capture immediate improvement needs, but omitted in A2, allowing a longer interval before reassessing qualitative feedback after the COVID-19 pandemic and assuming stable perceptions.

### Data collection

The survey was developed for completion by the physiatrist coordinating the rehabilitation team and/or by the head of the stroke unit, who were contacted and informed that the final dataset was to be analysed without identification of stroke units. Physiatrists were chosen as respondents for their central role in supervising rehabilitation plans, coordinating care across stroke phases, and their broad knowledge of local service organization. During A1, the questionnaire was sent by email to be filled out on paper and collected in person, by at least 2 of the authors, within 1 month, to maximize response rate. During A2, based on the high response achieved in A1, the questionnaire was delivered via Google Forms and the link emailed to each unit’s coordinating physiatrist.

### Statistical analysis

Descriptive statistics were used to summarize the general characteristics of the stroke units. Categorical variables were presented as absolute and relative frequencies, rounded to the nearest whole number. Data were analysed with Excel version 16.78 (Microsoft Corp, Redmond, WA, USA) and SPSS version 28.0.1.0 (IBM Corp, Armonk, NY, USA). Graphs were built with Excel version 16.78 (Microsoft Corp).

Temporal comparisons were based on data collected in A1 and A2. A descriptive, aggregate-level comparison between the timepoints was performed. Anonymization was used as a strategy to obtain more reliable and realistic responses.

## RESULTS

### Stroke units characteristics

At A1, 93% (*n* = 25 of 27) of stroke units responded and at A2, 80% (*n* = 28 of 35) responded. In A1, surveys were completed through the joint participation of the physiatrist and the head of the stroke unit (*n* = 21), by the head of unit alone (*n* = 3), or by the physiatrist alone (*n* = 1). In cases where both participated, any differences in responses were resolved through immediate discussion until consensus was reached.

In A2, surveys were completed by physiatrists alone (*n* = 28). Table I summarizes key indicators across both periods, allowing for an exploration of trends in stroke rehabilitation practices over time.

**Table I:** Survey results regarding Assessment 1 (2017–2018) and Assessment 2 (2023)

Domain Item			Assessment 1 (*n* = 25)		Assessment 2 (*n* = 28)	
			*n*	%	*n*	%
Multidisciplinary Team	Physiatrist dedicated to stroke unit	Yes	17	68%	22	79%
	Therapists dedicated to stroke unit	Yes	22	88%	22	79%
Coordination of Care	Weekly meeting of the stroke unit team	Yes	22	88%	25	89%
	Physiatrist in the stroke unit meeting	Yes	12	48%	15	54%
	Weekly meeting of the rehabilitation team	Yes	5	20%	20	72%
Early assessment and individualized rehabilitation planning	Average first-time observation by physiatrist – week	< 24 h	0	0%	2	7%
		24h–48 h	20	80%	22	79%
		48h–72 h	2	8%	2	7%
		> 72 h	0	0%	0	0%
		Not applicable^a^	3	12%	2	7%
	Average first-time observation time by physiatrist – weekend	< 24 h	0	0%	0	0%
		24h–48 h	0	0%	8	29%
		48h–72 h	17	68%	16	57%
		> 72 h	5	20%	2	7%
		Not applicable^a^	3	12%	2	7%
	Individualized therapeutic plan	Yes	23	92%	26	93%
Assessment of dysfunctions	Screening dysphagia	Yes	24	96%	25	89%
	Screening neurogenic bladder	Yes	2	8%	2	7%
Definition of post-discharge rehabilitation	Post-discharge rehabilitation plan	Yes	23	92%	25	89%
	Post-discharge provided independently by the DMT	Yes	15	60%	12	43%
	Does the physiatrist generally agree with DMT post-discharge plan?	Yes	12	48%	25	89%
		Not applicable^b^	0	0%	1	4%

Not applicable: (a) there was no physiatrist in the stroke unit; (b) the physiatrist did not provide post-discharge plan. There were no missing values.

### Rehabilitation care quality

**Fig. 1:**
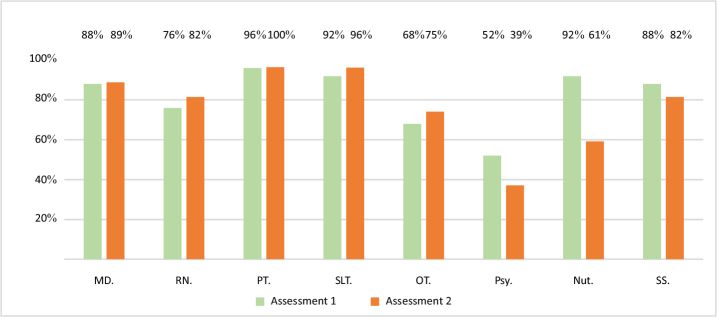
Multidisciplinary rehabilitation team members, in Assessment 1 (2017–2018) and Assessment 2 (2023). MD: medical doctor (physiatry), RN: rehabilitation nurse, PT: physiotherapist, SLT: speech and language therapist, OT: occupational therapist, Psy: psychologist, Nut: nutritionist, SS: social worker.

*Multidisciplinary Rehabilitation Team.* Fully rehabilitation constituted teams were present in 22% of the units in A1 and 18% in A2. Physiatrists were present in almost all units (88% and 89% for A1 and A2), though in 32% of stroke units in A1 and 21% of stroke units in A2 they were not specifically dedicated to stroke rehabilitation. The other team members with the highest presence were physiotherapists (96% and 100%), speech and language therapists (92% and 96%), and social workers (88% and 82%). Rehabilitation nurses (76% and 82%) and occupational therapists (68% and 75%) slightly increased their presence between the 2 assessment periods, while nutritionists’ presence declined (from 92% to 61%). Psychologists were often absent, in both assessment periods (52% and 39%) (Fig. 1).

### Coordination of care

Weekly stroke unit meetings occurred in 88% and 89% of units at A1 and A2. Physiatrists attended these in approximately half of the stroke units (48% and 54%). Weekly multidisciplinary rehabilitation team meetings rose sharply, from 20% of stroke units in A1 to 72% in A2 (see Table I).

### Early assessment and individualized rehabilitation planning

**Fig. 2:**
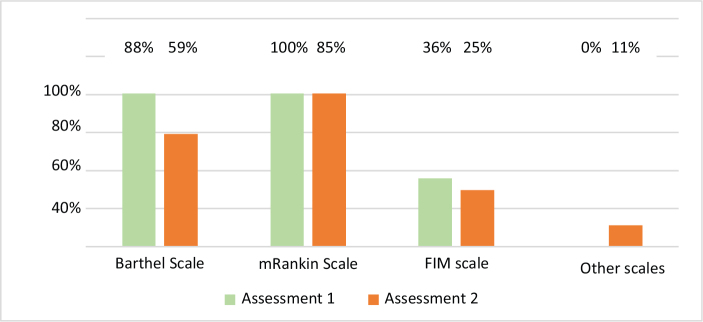
Scales used by the rehabilitation team in Assessment 1 (2017–18) and Assessment 2 (2023). Abbreviations: FIM: Functional Independence Measure. FOIS: Functional Oral Intake Scale (FOIS).

On weekdays, the initial rehabilitation assessment by the physiatrist was conducted within 48 h in 80% (A1) and 86% (A2) of stroke units. In contrast, weekend assessments within the same timeframe occurred in only 0% (A1) and 29% (A2) of units. Delayed assessments (> 72 h) were recorded in 20% and 7% of units during A1 and A2, respectively. Individualized therapeutic plans were common (92% of the units in both periods) (see Table I). The modified Rankin Scale (100% and 85% in A1 and A2) and the Barthel Index (88% and 59% in A1 and A2) were the most frequently used functional scales. Only 2 units reported systematically employing an additional scale (Functional Oral Intake Scale) in A2 (Fig. 2).

### Specific dysfunctions assessments

Systematic dysphagia screening was implemented in 96% of stroke units in A1 and 89% of stroke units in A2. Systematic neurogenic bladder assessment remained rare in both assessment periods (8% in A1 and 7% in A2) (see Table I).

### Post-discharge rehabilitation planning

Post-discharge care planning by the physiatrist was provided in 92% of stroke units in A1 and 89% in A2. However, in 60% of stroke units in A1 and 43% in A2, DMT independently determined post-discharge destinations within the context of Continued Care Units. Between periods, the agreement between physiatrists and DMT showed a substantial increase, from 48% in A1 to 89% in A2 (see Table I).

### Needed improvements

In A1, the most frequent need cited was for additional healthcare professionals (84%). Other areas included increasing outpatient rehabilitation treatment capacity in hospital facilities (36%), expanding subacute beds or dedicated inpatient rehabilitation wards (36%), extending physiatrist presence in stroke units (32%), providing more intensive rehabilitation treatment during stroke unit admission (28%), reducing transition waiting times to Continued Care Units or Rehabilitation Centres (24%), and improving access to additional rehabilitation support techniques, such as swallowing video fluoroscopy (8%).

## DISCUSSION

This study is the first to prospectively evaluate quality of stroke rehabilitation in Portuguese stroke units, over a 6-year period between 2017–2018 and 2023. The high response rate strengthens the reliability of these findings.

The multidisciplinary rehabilitation team is a vital component of stroke units (5, 6, 16, 26), yet in Portugal only about 20% of stroke units had fully staffed teams. Most units (≥ 75%) include a physiatrist, physiotherapist, speech and language therapist, rehabilitation nurse, and social worker, but 25% lacked a dedicated stroke rehabilitation team, despite the importance of specialization and timely intervention (18). Team composition varies globally (26–28); for instance, in Ontario, nearly all units include key therapists, nutritionists, and social workers (29), whereas a Spanish study reported widespread presence of physiatrists and physiotherapists but did not detail other professionals (28). In our data, team composition remained largely stable over time, with a moderate decline in nutritionists and psychologists and a slight rise in rehabilitation nurses, likely reflecting service reorganization during COVID-19. Such reductions require close monitoring, as prolonged shortages may compromise care quality.

Occupational therapy is strongly recommended in European guidelines (5, 6, 9, 15), yet over a third of Portuguese stroke units lack this element. Psychologists are also under-represented, despite guidelines recommending initial cognitive impairment and depression risk screenings (12, 18). These findings align with the Stroke Alliance for Europe report, which underscores a particular lack of occupational and psychological therapy across Europe (30). Portuguese stroke unit physicians identify workforce shortages as a major barrier to improving rehabilitation care. Sustained improvements will also depend on stable financing and strategic planning within the National Health Service, ensuring that guideline implementation is not compromised by short-term resource fluctuations or workforce pressures. Ensuring fully staffed multidisciplinary teams is paramount for delivering comprehensive care and achieving optimal rehabilitation outcomes. It is important to note that the ratios of all healthcare professionals to patients were not assessed. These ratios can significantly influence care quality; future research should include this across Portuguese stroke units.

Coordinated teamwork, a cornerstone of stroke units, is facilitated through regular meetings (6, 31), typically held weekly (26, 31). In Portugal, stroke unit and rehabilitation meetings are attended by physiatrists, following models described in the literature (31). While stroke unit meetings were in place in most stroke units across both evaluated periods, rehabilitation multidisciplinary meetings increased from 20% to 72%, reflecting improved coordination of stroke rehabilitation care. We hypothesize that local teams’ visits during first assessment may have raised awareness of the importance of regular meetings. These results are aligned with the Ontario Study Network study (29), the only study we found with comparable data regarding this issue. Effective communication and coordination are essential to optimize patient-centred rehabilitation care (6, 26, 32). Evaluating the quality and impact of these meetings on patient care could be a crucial next step in quality improvement. Future studies should include such assessments.

Early rehabilitation assessment and transition planning are both key quality indicators in stroke care (21). In Portugal, 75% of stroke units meet these targets within 48 h on weekdays compared with about 60% at weekends. These findings reflect the “weekend effect” observed in other studies, highlighting temporal variations in quality parameters (33, 34). Strengthening weekend rehabilitation efforts may therefore enhance healthcare quality and equity. To accomplish this, consistent standards of care should be maintained throughout the week, reducing disparities caused by fluctuating service levels and ensuring all people with stroke receive critical interventions when needed.

In Portugal, physiatrists conduct the initial rehabilitation assessment, evaluating patient function, safety, readiness, and ability to engage in therapy (5, 17). A high proportion of Portuguese stroke units reported implementing individualized rehabilitation plans and structured post-discharge rehabilitation planning. In contrast, in Greece, initial assessments are conducted by physiotherapists and occupational therapists, with reported compliance rates of 30–33% (27). In Australia, physiotherapists, occupational therapists, and speech and language therapists assess patients within 48 h in 79%, 65%, and 75% of cases, respectively (35). In Ontario, 72% of stroke unit teams offer early individualized plans and 62% conduct early post-discharge assessments (29). Although early discharge planning in Portugal had a generally good report, significant coordination issues with DMT were noted in many units during the first survey – largely because of differing views on rehabilitation planning. These issues have improved over the years, reflecting greater adherence to the Portuguese Standard Clinical Guideline for Stroke Rehabilitation. However, robust nationwide implementation of this guideline remains essential to ensure consistent, high-quality stroke care across the country.

Stroke unit physicians have identified an urgent need for more subacute beds and enhanced outpatient services post-discharge to expedite patient treatment. Expanding post-discharge rehabilitation capacity is essential – not only to complement improvements in acute stroke care but to ensure that the gains made are sustained. Without robust investment in subacute rehabilitation, the progress achieved in acute care could be lost.

Timely assessment of dysphagia assessment is widely recommended (5, 6, 9, 16, 20), ideally within 24 h of stroke onset. In Portugal, screening is well implemented (92% of stroke units), contrasting with lower rates in Greece (21.6%–41.6%) (27), China (78%) (36) and Belgium (< 50%) (37). Our data compares to the German Stroke Register Study Group (21) although their data were patient-level, retrospectively collated from electronic records, and ours refer to subjective report by the managers of the stroke units. In Portugal, delays occur because patients may remain in the emergency department for hours – or even days – before transfer to a stroke unit, postponing dysphagia screening. Future analyses should assess screening within 24 h of hospital admission, not only in stroke units. Implementing simple dysphagia screening protocols in emergency departments could further improve care. Additionally, inadequate neurogenic bladder screening underscores the need for national protocols and targeted training (9).

Functional scales such as the modified Rankin Scale (mRS), Barthel Index (BI), and Functional Independence Measure (FIM) are widely recommended for acute stroke rehabilitation (5, 6, 17, 38). In addition, many authors advocate for incorporating Patient-Reported Outcome Measures (PROMs) and Patient-Reported Experience Measures (PREMs) (39, 40). In Portuguese stroke units, the mRS is the predominant tool, followed by the BI, whereas the FIM is rarely used in the acute phase, likely due to its complexity and resource demands – even though rehabilitation centres place greater emphasis on completing this scale. Data on the prevalence of functional scales in acute stroke care is limited, though the mRS is the most frequently used in large studies. Notably, no Portuguese unit reported using PROMs or PREMs. Although clinical endpoints such as mortality or recurrence are essential, they do not fully reflect the lived impact of disabling but survivable strokes. Reliable and patient-centred markers are therefore crucial. In this regard, PROMs and PREMs merit further discussion, as their integration could enrich value-based healthcare by capturing patient perspectives on recovery and care quality. While the Registry of Stroke Care Quality (RES-Q) already provides real-time feedback via quality indicator dashboards, current efforts to integrate PROMs will be vital for capturing the patient’s voice (41). In Portugal, although 39 hospitals participate in RES-Q, they currently capture data for only 15% of stroke cases (41, 42), suggesting a significant opportunity to expand the registry’s role in benchmarking comprehensive rehabilitation outcomes.

This study has some limitations. One key limitation is the inability to match stroke unit responses between the 2 survey periods, which precluded a longitudinal analysis at the institutional level. Nonetheless, the descriptive comparison of aggregated data provides valuable insights into the evolution of rehabilitation practices and the implementation of national guidelines over a 6-year period. Our results rely on subjective reports from the heads of stroke units or coordinating physiatrists, with a potential bias from their desire to reflect favourable clinical practices. Furthermore, the perspectives of other multidisciplinary team members were not captured; this may have resulted in a fragmented view of daily clinical operations and interprofessional dynamics. Additionally, data collection differed between periods. In A1, responses were gathered in person after online distribution, with both unit coordinators and physiatrists contributing, when possible. In cases where their responses initially diverged, discussions were held immediately until consensus was reached. This approach ensured that all final responses reflected a shared and accurate representation of clinical practices within each unit. However, we acknowledge that this collaborative approach could potentially be influenced by hierarchical dynamics, where the perspective of the head of the unit might carry more weight. In A2, only physiatrists responded via email. These differences may have introduced bias when comparing indicators across periods. Although dual physician responses in A1 aimed to minimize self-report errors, all data were self-reported and unaudited, potentially affecting accuracy. Future studies on Portuguese adherence to quality indicators could complement subjective reports with real-world data. It is also essential to establish and evaluate these indicators across all stages of care – from acute to subacute and chronic phases – to ensure continuity and quality throughout. Leveraging national electronic health records and real-time registries could provide a more objective and continuous assessment of rehabilitation quality indicators, aligning Portugal with international trends in value-based healthcare and facilitating cross-country benchmarking. Finally, although our questionnaire was based on national and international guidelines, its structure may limit comparisons with studies from other countries. Developing a standardized European questionnaire could facilitate cross-country evaluations and comparisons.

In conclusion, Portuguese stroke units demonstrate moderate-to-high adherence to rehabilitation guidelines, with clear progress in multidisciplinary team communication, care, and discharge coordination over the past 6 years. Key strengths include early rehabilitation initiation and systematic dysphagia screening, but important gaps remain in full team staffing, weekend service coverage, bladder assessment, and standardized discharge planning.

Sustained investment in rehabilitation – on a par with acute stroke care – is essential to consolidate these gains, expand access, and ensure patient-centred, high-quality recovery for all stroke survivors

## Supplementary Material


