# Supplementary material for: QUALITY OF REHABILITATION CARE IN PORTUGUESE STROKE UNITS: FINDINGS FROM 2017–2018 AND 2023 NATIONAL CROSS‑SECTIONAL SURVEYS
Source: J Rehabil Med. 2026 Mar 29;58:44855. doi: 10.2340/jrm.v58.44855 (PMC13044904; doi:10.2340/jrm.v58.44855)
Supplement: Supplementary file 1 [file JRM-58-44855-s1.pdf]

Supplementary material has been published as submitted. It has not been copyedited, or typeset by Journal of rehabilitation Medicine

Table SI. Survey to collect data on characteristics of stroke unit rehabilitation care in Portuguese stroke units.

**Quality indicator**	**Questions**
Structure	1. Constitution and characterization of the Multidisciplinary Team<br>a. Presence of physiatrist? Yes – No<br>b. Presence of physiotherapist? Yes – No<br>c. Presence of speech and language therapist? Yes – No<br>d. Presence of occupational therapist? Yes – No<br>e. Presence of social worker? Yes - No<br>f. Presence of nutritionist? Yes - No<br>g. Presence of (neuro)psychologist? Yes – No
Structure	2. Is the physiatrist dedicated to stroke rehabilitation care? Yes – No
Structure	3. Are the therapists dedicated to stroke rehabilitation care? Yes – No
Process	4. Coordination of care through meetings<br>a. Is there at least a weekly meeting of the stroke unit team? Yes – No<br>b. Is the physiatrist regularly present at this meeting? Yes – No<br>c. Is there at least a weekly meeting of the rehabilitation stroke team? Yes – No
Process	5. Early clinical and functional assessment and individualized therapeutic planning<br>a. What is the average time for the first assessment by physiatry at weekdays?<br>< 24h<br>24 – 48h<br>48 – 72h<br>> 72h<br>b. What is the average time for the first assessment by physiatry at weekends?<br>< 24h<br>24 – 48h<br>48 – 72h<br>> 72h<br>c. Is an individualized therapeutic plan developed for each patient? Yes – No<br>d. Which functional scales are used? Modified Rankin Scale Yes - No<br>Barthel Index Yes – No<br>Functional Independence Measure Yes – No<br>e. Please write other scales routinely used by rehabilitation staff.
Process	6. Specific assessment of dysfunctions – dysphagia and neurogenic bladder<br>a. Is dysphagia screening routinely performed? Yes – No<br>b. Is neurogenic bladder routinely performed? Yes – No
Process	7. Definition of post-discharge rehabilitation treatment<br>a. Is discharge planning and definition of post-discharge rehabilitation treatment carried out by the physiatrist? Yes – No<br>b. Can discharge planning and the definition of post-discharge rehabilitation treatment be carried out independently by the Discharge Management Teams in your hospital? Yes – No<br>c. Does the physiatrist generally agree with the recommendations proposed by the Discharge Management Teams?

Open question: What changes do you consider most important to improve rehabilitation care in your stroke unit?
